# Microcurrent Stimulation Triggers MAPK Signaling and TGF-β1 Release in Fibroblast and Osteoblast-Like Cell Lines

**DOI:** 10.3390/cells9091924

**Published:** 2020-08-19

**Authors:** Evangelia Konstantinou, Zoi Zagoriti, Anastasia Pyriochou, Konstantinos Poulas

**Affiliations:** Laboratory of Molecular Biology and Immunology, Department of Pharmacy, University of Patras, 26504 Rio, Greece; ekonstan@upatras.gr (E.K.); zoizag@upatras.gr (Z.Z.); apyriohou@upatras.gr (A.P.)

**Keywords:** electrical stimulation, fibroblasts, osteoblast-like cells, signaling pathways

## Abstract

Wound healing constitutes an essential process for all organisms and involves a sequence of three phases. The disruption or elongation of any of these phases can lead to a chronic or non-healing wound. Electrical stimulation accelerates wound healing by mimicking the current that is generated in the skin after any injury. Here, we sought to identify the molecular mechanisms involved in the healing process following in vitro microcurrent stimulation—a type of electrotherapy. Our results concluded that microcurrents promote cell proliferation and migration in an ERK 1/2- or p38-dependent way. Furthermore, microcurrents induce the secretion of transforming growth factor-beta-1 (TGF-β1) in fibroblasts and osteoblast-like cells. Interestingly, transcriptomic analysis uncovered that microcurrents enhance the transcriptional activation of genes implicated in Hedgehog, TGF-β1 and MAPK signaling pathways. Overall, our results demonstrate that microcurrents may enhance wound closure through a combination of signal transductions, via MAPK’s phosphorylation, and the transcriptional activation of specific genes involved in the healing process. These mechanisms should be further examined in vivo, in order to verify the beneficial effects of microcurrents in wound or fracture healing.

## 1. Introduction

The ability of an organism to respond to injury, in order to achieve tissue repair and to maintain homeostasis, is of high significance [1,2]. Acute wounds normally heal in a steady and efficient way, consisting of three distinct but overlapping phases: inflammation, tissue formation and remodeling [1,2,3,4,5]. In each phase, different cell types, such as macrophages, neutrophils, keratinocytes, fibroblasts, endothelial cells etc., are recruited and interact with each other to restore the damaged tissue area [1,3,6,7,8]. Impairment in one or more of these phases leads to delayed wound healing or to a chronic, non-healing wound [9,10]. In chronic wounds, parts of the injured areas are found in different stages of healing or are “trapped” in a certain phase for a prolonged period [3,9]. However, the molecular mechanisms that lead to ineffective wound healing are not clearly elucidated.

When a skin injury occurs, a flow of current, immediately, generates an electric field through the wound, known as “the current of injury”, which activates and recruits the cell types involved in the healing process, to the site of the wound [5,11,12,13]. These electric fields (EFs) stimulate division, proliferation and the directional migration of cells, in order to initiate the wound healing process [13,14,15]. Electrical stimulation (ES) has been introduced as a treatment for chronic wounds, as it mimics the “current of injury” and has beneficial effects in every phase of wound healing [6,16,17,18]. ES has been shown to promote the migration of keratinocytes and macrophages, to stimulate fibroblasts and to induce DNA synthesis, extracellular matrix (ECM) deposition, and the expression of collagen, growth factors (vascular endothelial growth factor (VEGF), transforming growth factor-β (TGF-β)) and receptors (epidermal growth factor receptor (EGFR) and vascular endothelial growth factor receptor (VEGFR)) [5,6,14,17,19,20].

In recent years, ES of low amplitude and frequency has become an increasingly popular and effective method for the treatment of chronic wounds on the soft skin or at the bones [18,21]. Wireless microcurrent stimulation (WMCS) is a form of ES applied remotely to the wound. This technology is easy to handle, non-traumatic, painless and it entails no risk of infection, due to the absence of physical contact [15,16,22,23,24]. WMCS generates microcurrents by spraying charged atmospheric gases to the injured tissue, while an adaptable bracelet (neutral electrode) worn around the healthy wrist or ankle of the individual, creates a closed circuit without any contact with the wound [22]. Clinical studies have shown complete repair in patients with chronic wounds within 30 days of microcurrents’ exposure [16,22]. However, the underlying molecular mechanisms that enhance wound and fracture healing, remain unspecified.

In the present study, we intended to investigate the effects of microcurrents in fibroblasts and osteoblast-like cells. For this purpose, WMCS was applied as a method to generate microcurrents and deliver ES remotely to the cell culture. Initially, we examined whether stimulation with microcurrents could affect the signaling pathways mediated by the mitogen-activated protein kinases (MAPKs) ERK 1/2 and p38, which are involved in many cellular functions, i.e., cell proliferation and migration [25]. Furthermore, we sought to determine microcurrents-induced TGF-β1 release, which is associated with an increased synthesis of collagen I, III, IV, fibronectin and other ECM components during the healing process [19]. Finally, transcriptomic analysis was performed in osteoblasts, in order to identify the potential differences in the expression profile of osteoblasts under microcurrents treatment. Overall, this study attempted to describe a molecular network of genes and signaling pathways activated upon microcurrent stimulation in cell types involved in the healing process.

## 2. Materials and Methods

### 2.1. Cell Culture

NIH3T3 and MG-63 cells (ATCC, Rockville, MD, USA) were cultured in Dulbecco’s modified Eagle’s medium (DMEM, Biosera, Nuaillé, France), supplemented with 10% fetal bovine serum (FBS, Gibco, Thermo Fisher Scientific, Waltham, MA, USA) and 1% penicillin/streptomycin (Biosera). Cells were cultured without any stimulatory supplements or additional vitamins and were incubated at 37 °C in a 5% CO_2_ humidified atmosphere. For passaging, the cells were detached with trypsin/ethylenediaminetetraacetic acid (EDTA) (Biosera) and subsequently re-plated. The cells were routinely subjected to mycoplasma testing and found to be negative.

### 2.2. Generation of Microcurrents

For the generation of microcurrents, the commercially available WMCS W200 device (Wetlinghealth, Fredensborg, Denmark) was used. To avoid the loss of microcurrents, the stimulation of cells was performed in glass cell culture dishes. Regarding the setting up of the experiment, the return wire of the device was placed under the glass cell culture dish and the treatment head of the device was positioned in the appropriate distance from the dish (approximately 10–12 cm from the bottom of the plate), until the continuous beeping sound stopped emitting (Supplementary Materials, Figure S1A). This warning sound occurs when the distance is too great and the transferred microcurrent does not reach the target. The thickness of the culture medium was 3 mm and the electric conductivity of DMEM was determined to be ~1.6 S/m [26,27]. The required microcurrent amount and duration of the treatment were set in the control panel. For our experiments, the amplitude of the applied currents was constantly at 2 μA. During the exposure, the pH and the temperature of the culture medium were monitored by using a pH-meter and an infrared thermometer.

### 2.3. Western Blot Analysis

NIH3T3 and MG-63 cells in ~90% confluency were starved in a serum-free culture medium for 4 h and then stimulated with microcurrents for 5, 10, 20 and 30 min, corresponding to the transfer of −414, −916, −1672 and −3100 microcoulomb (μC) O_2_^−^, respectively. The cells were immediately lysed in 120 μL SDS sample buffer (0.16 M Tris-HCl pH 6.8, 4% SDS, 20% glycerol, 100 mM dithiothreitol (DTT), and 0.01% bromophenol blue). Equal protein amounts were resolved on 4–12% Bis-Tris gels followed by Western blot analysis onto polyvinylidene difluoride membranes (Macherey Nagel GmbH & Co. KG, Düren, Germany). Membranes were blocked with 5% milk diluted in 0.1% PBS-Tween at room temperature for 1 h. Then, the membranes were incubated overnight at 4 °C with primary antibodies for the phosphorylated forms of ERK 1/2 (1:2000 in 5% BSA 0.1% PBS-Tween, Cell Signaling, Beverly, MA, USA), p38 (1:1000 in 5% BSA 0.1% PBS-Tween, Cell Signaling) total-ERK 1/2 (1:1000 in 5% BSA 0.1% PBS-Tween, Origene Technologies, USA), total-p38 (1:3000 in 5% BSA 0.1% PBS-Tween, Origene Technologies, USA) and β-actin (1:1000 in 5% BSA 0.1% PBS-Tween, Cell Signaling) as a loading control. Membranes were washed with 0.1% PBS-Tween and incubated for 1 h at room temperature with goat anti-rabbit secondary antibody conjugated with horseradish peroxidase (1:2000 in 5% milk 0.1% PBS-Tween, Cell Signaling), and the immunoblots were detected by an enhanced chemiluminescence (ECL) detection system (Thermo Fischer Scientific) and X-Ray film (Kisker Biotech GmbH & Co. KG, Steinfurt, Germany). For the blockage of ERK 1/2 phosphorylation, NIH3T3 and MG-63 cells were incubated with 10 μM of U0126 (Cell Signaling) for 30 min prior to stimulation. For phospho-p38 inhibition, the cells were treated with 10 μM SB203580 (Cell Signaling) inhibitor for 1 h prior to stimulation. The cell lysates were collected and analyzed as previously described. For the validation of ERK 1/2 and p38 blockage, the membranes were incubated with anti-phospho-ERK 1/2 antibody (1:2000 in 5% BSA 0.1% PBS-Tween, Cell Signaling) and with antibody against phospho-MAPKAPK-2, which is a direct target of phospho-p38 (1:1000 in 5% BSA 0.1% PBS-Tween, Cell Signaling), respectively. Dimethyl sulfoxide (DMSO) was used as negative control.

### 2.4. Cell Viability Assay

Mitochondrial activity, and by extension, cell viability, were determined by the established MTT (3-[4–Dimethylthiazol-2-yl]-2,5-diphenyltetrazolium bromide) chromogenic assay. NIH3T3 and MG-63 were plated in 9 cm^2^ cell culture plates (3 × 10^4^ cells per plate). Twelve hours after seeding, the cells were serum starved and then stimulated with microcurrents until −414, −916, −1672 and −3100 μC O_2_^−^ were transferred. The cells were incubated at 37 °C in 5% CO_2_ for 24 h. In the culture medium of all experimental and control cells, 150 μL of MTT solution (5 mg/mL in PBS) were added and after 4 h of incubation, formazan crystals were solubilized by the addition of 300 μL of dimethyl sulfoxide (DMSO) (Applichem GmbH, Darmstadt, Germany). Formazan dye was quantified spectrophotometrically at 492 nm. Cell viability results were calculated as the percentages of control cells.

### 2.5. In Vitro Cell Migration Assay

Cells were grown to confluency in 78.5 cm^2^ plates and were serum starved for 4 h. Cells were incubated with inhibitors U0126 or SB203580, in a final concentration of 10 μM, for 30 min and 1 h prior to stimulation (transfer of −916 μC O_2_^−^ to NIH3T3 and −414 μC O_2_^−^ to MG-63 cells), respectively. A straight scratch was created using a P200 pipette tip. The cells were then washed three times with PBS, and further cultured in full medium. The gap width of scratch re-population was measured and recorded after 0, 24, 48 and 72 h.

### 2.6. Cell Proliferation

The cells were plated in 9 cm^2^ cell culture plates (5 × 10^4^ cells per plate) and incubated at 37 °C in 5% CO_2_ for 12 h. Then, the cells were serum starved for 4 h and treated with 10 μM U0126 or 10 μM SB203580, or with the combination of both inhibitors, prior to the stimulation with microcurrents (stimulation lasted until −916 μC O_2_^−^ were transferred to NIH3T3 cells and −414 μC O_2_^−^ to MG-63). The cells were detached with trypsin and viable cells were counted on a Neubauer chamber, 24 and 48 h following stimulation.

### 2.7. Quantitation of TGF- β1 Release

Release of biologically active TGF-β1 was quantitated using a commercially available immunoassay kit, according to the manufacturer’s instructions (Human/Mouse TGF beta 1 Uncoated ELISA, Invitrogen, Thermo Fisher Scientific). Briefly, the supernatants from NIH3T3 and MG-63 cell cultures were collected at 4, 6, 8, 24 and 48 h after transferring −916 μC O_2_^−^ to NIH3T3 cells and −414 μC O_2_^−^ to MG-63. The same procedure was followed after the treatment with U0126 or SB203580 or both inhibitors in both cell lines. Cytokine levels were determined by means of triplicated measurements and optical density was measured at 450 nm (MPR 700 Plate Reader). Results were standardized by using internal controls supplied with the kit, with a known concentration of the target protein.

### 2.8. RNA Extraction and cDNA Synthesis

Total RNA was extracted from the control and stimulated NIH3T3 and MG-63 cells at different time points after the exposure to microcurrents (1.5, 2 and 3 h after the exposure of NIH3T3 to −916 μC O_2_^−^ and 8 h after the exposure of MG-63 cells to −414 μC O_2_^−^), using Nucleospin RNA kit (Macherey Nagel GmbH & Co. KG, Düren, Germany). RNA concentration and purity were determined by measurement in Q6000 UV-Vis Spectrophotometer (Quawell) and by agarose gel electrophoresis to assess the integrity of 18S and 28S rRNA bands. RNA was reverse transcribed to cDNA using PrimeScript first strand cDNA Synthesis Kit (Takara Bio Inc., Shiga, Japan), according to the manufacturer’s instructions.

### 2.9. RNA Sequencing

#### 2.9.1. Next Generation Sequencing

Total RNA was measured in NanoDrop (ND1000 Spectrophotometer, PEQLAB, Erlangen, Germany). The samples were diluted accordingly to a mean concentration of approximately 100–150 ng/μL and their quality assessed in a Bioanalyzer (Agilent Technologies, Santa Clara, CA, USA) using the Agilent RNA 6000 Nano Kit reagents and protocol (Agilent Technologies, Santa Clara, CA, USA) [28]. For the library preparation, the 3′ mRNA-Seq Library Prep Kit Protocol for Ion Torrent (QuantSeq-LEXOGEN Vienna, Austria) was used according to the manufacturer’s instructions. Up to 500 ng of RNA was used for the first and second strand synthesis, followed by 13 cycles of amplification. Library quality and quantity were assessed in Bioanalyzer using the DNA High Sensitivity Kit reagents and protocol (Agilent Technologies, Santa Clara, CA, USA). The quantified, barcoded libraries were pooled together at a final concentration of 7pM. The pools were templated and enriched on an Ion Proton One Touch system. Templating was performed using the Ion PI Hi-Q OT2 200 Kit (Thermo Fischer Scientific), followed by sequencing with the Ion PI Hi-Q Sequencing 200 Kit on Ion Proton PI V2 chips (Thermo Fischer Scientific) according to the commercially available protocols, on an Ion Proton System, according to the manufacturer’s instructions.

#### 2.9.2. Short Read Mapping

The obtained FASTQ files were mapped on the UCSC hg19 reference genome using a two-phase mapping procedure. Firstly, the short reads were mapped using tophat2, with default settings and using additional transcript annotation data for the hg19 genome from Illumina iGenomes [29,30]. Then, the reads which remained unmapped were submitted to a second round of mapping using Bowtie2 with the --local and --very-sensitive local switches turned on.

#### 2.9.3. Differential Expression Analysis

Differential expression analysis was performed using the Bioconductor package metaseqR [31]. The BAM files obtained after short read mapping, one for each RNA-Seq sample, were summarized to a 3′UTR read counts table, using the Bioconductor package GenomicRanges. In the final read counts table, each row represented each column one RNA-Seq sample and each cell, the corresponding read counts associated with each row and column. The gene counts table was normalized for inherent systematic or experimental biases (e.g., sequencing depth, gene length, GC content bias etc.) using the Bioconductor package DESeq after removing the genes that had zero counts over all the RNA-Seq samples [31,32]. Prior to the statistical testing procedure, the gene read counts were filtered for possible artifacts that could affect the subsequent statistical testing procedures. Genes presenting any of the following were excluded from further analysis: (i) genes with a total length less than 500, (ii) genes whose average reads per 100 bp was less than the 25th quantile of the total normalized distribution of average reads per 100 bp, (iii) genes with read counts below the median read counts of the total normalized count distribution, (iv) genes whose Ensembl biotype matched the following: rRNA, TR_V_pseudogene, TR_J_pseudogene, IG_C_pseudogene, IG_J_pseudogene, IG_V_pseudogene, and (v) the genes where 50% of the samples did not present more than five normalized counts across all samples [31,32]. The resulting gene counts table was subjected to differential expression analysis for the contrasts WM (cells stimulated with wireless microcurrents) vs. the Ctrl. (control cells), using a combination of the Bioconductor packages DESeq, edgeR, limma, NBPSeq and NOISeq. In order to combine the statistical significance from the multiple algorithms, the PANDORA weighted *p*-value across the results method was calculated and applied [33].

### 2.10. Real-Time PCR

Real-time PCR was carried out using KAPA SYBR Fast Master Mix (Kapa Biosystems, Wilmington, MA, USA) on the LightCycler 96 Instrument (Roche, Basal, Switzerland). Real-time PCR reactions were performed in a total volume of 15 μL, containing 12.5 ng of cDNA, 7.5 μL KAPA SYBR Fast Master Mix, primers in a final concentration of 200 nM and ddH_2_O. Cycling conditions were as follows: 95 °C for 3 min, 40 cycles of denaturation at 95 °C for 3 s and amplification at 60 °C for 30 s and a final melting curve analysis. *Hypoxanthine phosphoribosyltransferase* (*HPRT*) was used as a housekeeping gene for the normalization of gene expression levels. Relative gene expression was calculated by the 2^−ΔΔCt^ method. Primer sequences were designed using the NCBI Primer-Blast designing tool (Table S4).

### 2.11. Statistical Analysis

Data are expressed as the means ± standard error of the mean (SEM) of at least three independent experiments. The statistical analysis was performed using one-way ANOVA. A value of *p* < 0.05 was considered statistically significant.

## 3. Results

### 3.1. Stimulation with Microcurrents Activates ERK 1/2 and p38 MAP Kinases

**Figure 1:**
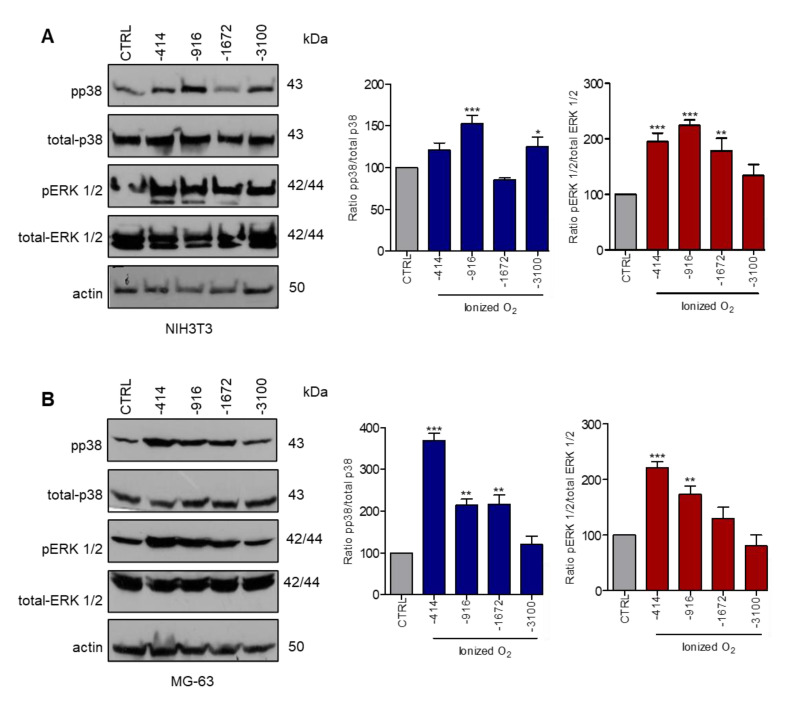
Treatment with microcurrents activates ERK 1/2 and p38 in mouse fibroblasts NIH3T3 and human osteoblast-like MG-63 cells. Total cell lysates from (**A**), NIH3T3 and (**B**), MG-63 cell cultures were separated by SDS-PAGE and immunoblotted to detect the phosphorylation levels of ERK 1/2 and p38. Graphs depict the phosphorylation levels of ERK 1/2 and p38 normalized to total-ERK 1/2 and total p38, respectively. Actin was used as the loading control. (* *p* < 0.05, ** *p* < 0.01, *** *p* < 0.005, treated vs. control, *N* = 3).

To identify whether the microcurrents activate specific signaling pathways in mammalian cells, we examined the phosphorylation of ERK 1/2 and p38 kinases in two different cell lines: NIH3T3 and MG-63. NIH3T3 cells are mouse embryonic fibroblasts, which participate in all three phases of wound healing by mediating several important activities for wound closure [34,35]. Osteoblasts are involved in fracture healing. Therefore, MG-63 were chosen as osteosarcoma cells sharing certain osteoblastic features [36,37]. NIH3T3 and MG-63 cell cultures were serum starved and subsequently exposed to microcurrents (Figure S1A) until different charges of ionized O_2_ of −414, −916, −1672 and −3100 μC were transferred (Figure 1A,B). Treatment with microcurrents had no cytotoxic effect and did not induce changes in the temperature and pH of the culture medium, as shown in Figure S1B–D. Protein extracts were collected and analyzed using specific antibodies for the phosphorylated forms of ERK 1/2 and p38. As shown in Figure 1A, the maximum phosphorylation of ERK 1/2 and p38 in NIH3T3 cells was evident when −916 μC O_2_^−^ were transferred. Regarding the MG-63 cells (Figure 1B), higher levels of ERK 1/2 and p38 phosphorylation were detected following the transfer of −414 μC O_2_^−^ and started to decline afterwards. Taken together, these data suggest that the microcurrent stimulation activates MAPKs ERK 1/2 and p38, via phosphorylation, in osteoblasts and fibroblasts, following the transfer of −414 μC and −916 μC of O_2_^−^, respectively.

### 3.2. Microcurrents Induce Wound Closure in an ERK 1/2- or p38-Dependent Manner In Vitro

**Figure 2:**
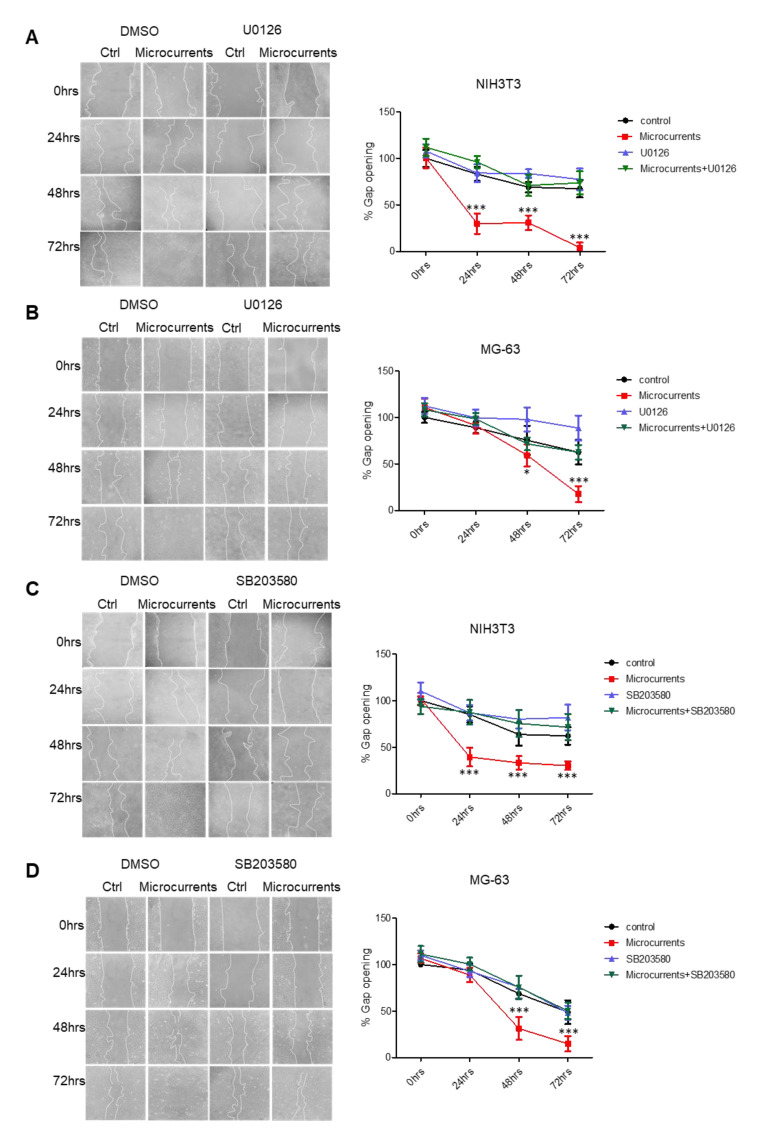
Stimulation with microcurrents accelerates wound closure in fibroblasts and osteoblast-like cells. (**A**,**B**) Representative images of a wound healing assay in NIH3T3 and MG-63 cells stimulated with microcurrents, in the presence and absence of an U0126 inhibitor. Quantification of the wound area (gap opening) was performed every 24 h. (**C**,**D**), Representative images of wound healing assay in NIH3T3 and MG-63 cells treated with microcurrents, in the presence and absence of p38 inhibitor, SB203580. Graphs represent the quantification of the gap opening in different time points. Microscopy images recorded under identical conditions with a 100× magnification are shown. (* *p* < 0.05, *** *p* < 0.005, *N* = 3, *n* = 3, microcurrent stimulation vs. control).

To directly examine the effects of microcurrent stimulation on the healing process, wound closure was monitored in monolayer cultures. For this purpose, the scratch wound assays were performed in NIH3T3 and MG-63 cells and the rate of gap closure was determined upon stimulation with microcurrents. The percentage of wound closure was measured daily until the surface of the wound had been fully “healed”. When the microcurrents were applied and the optimal number of electric charges was transferred (−916 μC O_2_^−^ for NIH3T3 and −414 μC O_2_^−^ for MG-63), both NIH3T3 (Figure 2A,C) and MG-63 cells (Figure 2B,D) showed increased migration and proliferation rates compared to the untreated cells (control). As a result, the stimulation with microcurrents enhances the wound closure in NIH3T3 and MG-63 cells. In order to investigate whether microcurrent-dependent wound closure requires MAPKs ERK 1/2 or p38 activation, we repeated the experiments, in the presence of inhibitors, U0126 for ERK 1/2 or SB203580 for p38. Treatment with ERK 1/2 or p38 inhibitor in stimulated NIH3T3 and MG-63 cells caused reduced wound closure rate (Figure 2A–D). These results indicate the significance of ERK 1/2 or p38 MAPKs activation during wound closure induced by microcurrents. To validate the specificity of the inhibitors, U0126 and SB203580 regarding the blockage of ERK 1/2 or p38 activation, we analyzed the protein extracts from NIH3T3 and MG-63 cells, treated with U0126 or SB203580 and stimulated with microcurrents. The analysis revealed that the inhibitors U0126 and SB203580 blocked MAPKs’ phosphorylation, both in the untreated and in the microcurrent-treated cells (Figure S2A,B). In general, our results demonstrate that stimulation with microcurrents induces cellular migration and/or proliferation through the activation of ERK 1/2 or p38 MAPKs, leading to enhanced wound closure.

### 3.3. Microcurrents Enhance Cell Proliferation through ERK 1/2 and p38 Activation

**Figure 3:**
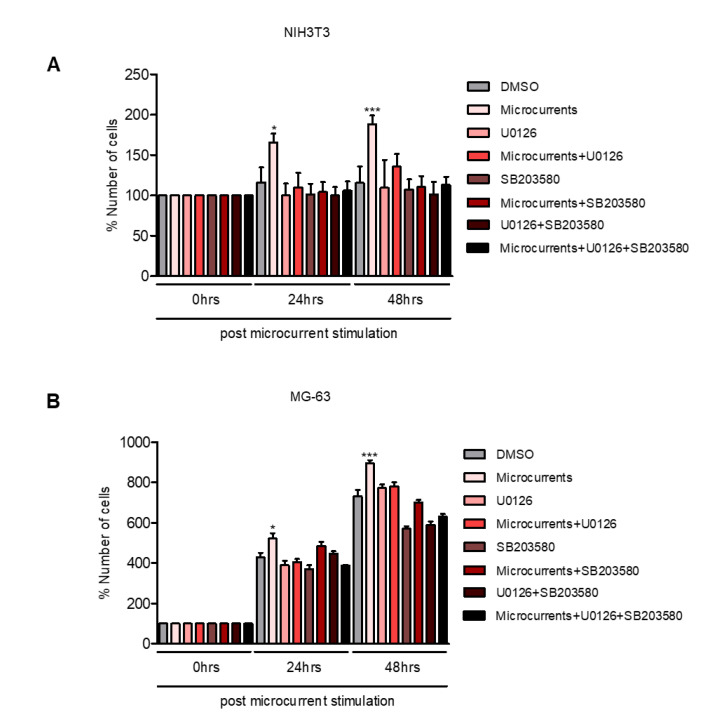
Microcurrent stimulation enhances the cellular proliferation rate in the fibroblasts and osteoblasts. (**A**) Graph depicts the proliferation rate of NIH3T3 cells, after the stimulation with microcurrents, in the presence and absence of U0126 or SB203580 or both inhibitors, in different time periods. (**B**) Graphical representation of the proliferation rate of MG-63 cells, following the stimulation with microcurrents and the treatment with U0126 or SB203580 or both inhibitors. (* *p* < 0.05, *** *p* < 0.005, *N* = 3, *n* = 3, microcurrent stimulation vs. control).

Cellular migration and proliferation are crucial events of the wound healing process [3]. To test whether stimulation with microcurrents could enhance cell proliferation, we determined the proliferation rate of NIH3T3 and MG-63 cells, in response to treatment with microcurrents. NIH3T3 and MG-63 cells were serum starved and then stimulated with microcurrents. The number of cells was determined 24 and 48 h following the stimulation with microcurrents. The analysis revealed that microcurrents significantly increased the proliferation rate of both NIH3T3 and MG-63 cells. Moreover, in the presence of an ERK 1/2 or p38 inhibitor (U0126 and SB203580, respectively), or both the inhibitors, the microcurrent-induced proliferation was abolished (Figure 3A,B). Overall, these data suggest that the stimulation with microcurrents enhances cellular proliferation in an ERK 1/2- and p38-dependent way, as examined in the cell types participating in wound healing.

### 3.4. Treatment with Microcurrents Increases TGF-β1 Secretion

**Figure 4:**
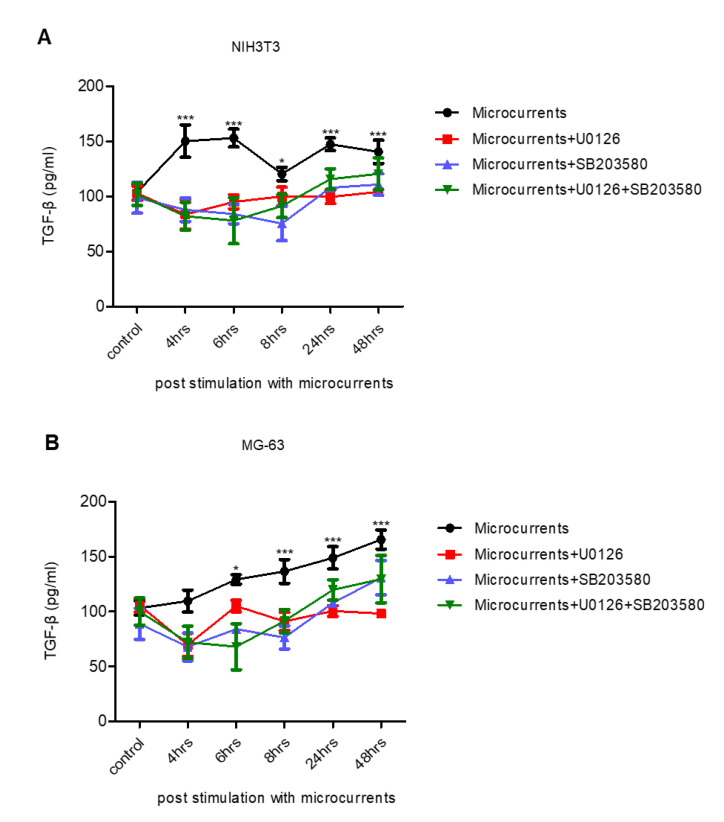
Fibroblasts and osteoblasts secrete TGF-β1 following stimulation with microcurrents. Quantification of TGF-β1 concentration in the supernatants from (**A**) NIH3T3 and (**B**) MG-63 cells stimulated with microcurrents, in the presence and absence of U0126 or SB203580 or the combination of both inhibitors, using ELISA. (* *p* < 0.05, *** *p* < 0.005, *N* = 3, *n* = 3, microcurrent stimulation vs. control).

During the healing process, cells interact with various ECM components, such as collagens, fibronectin, proteoglycans, etc. This process is mediated by cytokines and growth factors [38]. TGF-β1 is an essential component for matrix formation, as it stimulates the synthesis of matrix proteins, such as collagen I, and the receptors associated with these proteins [19]. Our goal was to investigate whether microcurrents could induce TGF-β1 secretion, thus contributing to the wound healing process. For this reason, TGF-β1 levels were determined, using ELISA, in the supernatants from NIH3T3 and MG-63 cell cultures stimulated with microcurrents (−916 μC O_2_^−^ were transferred to NIH3T3 and −414 μC O_2_^−^ to MG-63). In more detail, TGF-β1 levels were measured at 4, 6, 8, 24 and 48 h after stimulation (Figure 4A,B). As depicted in Figure 4A, TGF-β1 levels were elevated in the supernatants from stimulated NIH3T3 cells compared to the control. Furthermore, TGF-β1 levels were increased in the supernatants collected from MG-63 cells, at 6, 8, 24 and 48 h post-treatment with microcurrents (Figure 4B). To unravel the possible implication of ERK 1/2 and p38 kinases in TGF-β1 release upon stimulation with microcurrents, we repeated the experiments using the U0126, SB203580 or the combination of both inhibitors. Treatment with the ERK 1/2 and p38 inhibitors blocked the TGF-β1 release in the microcurrent-stimulated NIH3T3 (Figure 4A) and MG-63 cells (Figure 4B). In summary, these data suggest that microcurrents enhance the secretion of TGF-β1 in an ERK 1/2- and p38-dependent manner, which may contribute to the healing process through the synthesis of ECM components.

### 3.5. Upregulation of Genes Participating in TGF-β, MAPK and Hedgehog Signaling Pathways upon Stimulation with Microcurrents

**Figure 5:**
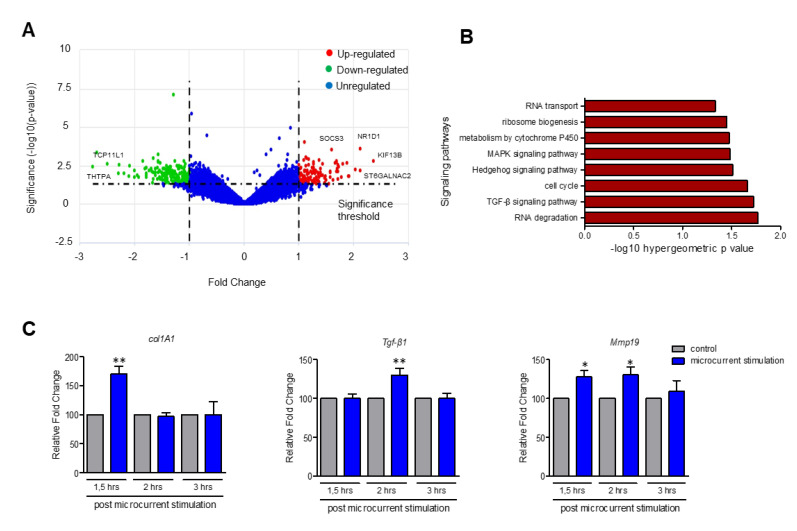
Stimulation with microcurrents activates the expression of genes participating in the TGF-β, MAPK and Hedgehog pathways. (**A**) Analysis of RNA seq data depicted by volcano plot, showed that among the 1139 statistically significant genes, 121 were upregulated, 202 were downregulated and 816 were not differentially expressed. (**B**) Gene ontology analysis of upregulated genes using GeneCodis, revealed that these genes were implicated in TGF-β, MAPK and Hedgehog signaling pathways, as well as in the progression of the cell cycle. (**C**) Real-time PCR analysis for wound healing-related genes *Tgf-β1*, *Col1a1* and *Mmp19* in NIH3T3 cells. The mRNA levels for all genes were determined 1.5, 2 and 3 h post-stimulation in NIH3T3 cells. *HPRT* was used as the reference gene (* *p* < 0.05, ** *p* < 0.01, *N* = 3, *n* = 3, microcurrent stimulation vs. control).

To decipher the total transcriptomic profile of MG-63 cells, following the treatment with −414 μC O_2_^−^, RNA seq was applied to the total RNA extracted from the stimulated and control cells at 8 h post stimulation. The RNA seq data analysis revealed that 202 genes were downregulated, 121 were upregulated, while 816 genes did not show significant differences in the expression levels between the stimulated and control cells (Figure 5A and Figure S3A,B, Tables S1 and S3). Gene ontology analysis revealed that several upregulated genes participate in cellular processes, mediated by TGF-β, MAPK and Hedgehog signaling pathways, as well as in cell cycle progression (Figure 5B and Table S2). On the other hand, 25 of the 202 downregulated genes were found to participate in metabolic pathways, with five of them, e.g., *ENTPD6: ectonucleoside triphosphate diphosphohydrolase 6* and *PDE5A*: *phosphodiesterase 5A*, implicated in the metabolism of purine (Figure S4 and Table S2). Real-time PCR was performed to verify the differences in the gene expression of selected target genes identified by RNA seq analysis. As shown in Figure S5A, *NR1D1*, *KIF13b* and *ST6GALNAC2* were highly expressed in the stimulated compared to the control MG-63 cells. Furthermore, we examined the expression levels of *Tgf-β1*, *Col1A1* and *Mmp19* in NIH3T3 cells, treated with −916 μC O_2_^−^. As shown in Figure 5C, *Col1A1* expression was significantly higher at 1.5 h post stimulation compared to the control, while expression levels of *Tgf-β1* were significantly increased at 2 h after stimulation. *Mmp19* expression was significantly elevated both at 1.5 and 2 h following stimulation. The expression levels of these genes started to decline in the samples collected 3 h post-stimulation (Figure 5C). Hedgehog (Hh) signaling pathway is implicated in wound healing through the activation of cell proliferation and angiogenesis [39,40,41]. To investigate the potential activation of the Hh signaling pathway upon microcurrent stimulation in NIH3T3 cells, the expression levels of *Smo*, *Ptch1*, *Gli3* were examined by real-time PCR. The analysis revealed that *Smo*, *Ptch1*, *Gli3* genes were upregulated in stimulated compared to control cells (Figure S5B). To sum up, these data suggest that stimulation with microcurrents leads to the transcriptional activation of multiple genes participating in MAPK-, Hedgehog- or TGF-β1-signaling pathways.

## 4. Discussion

Wound healing is a very efficient process, which starts immediately after the tissue has been injured [4]. Chronic ulcers—occurring when the healing process is disturbed—constitute a substantial socioeconomic burden, as they may lead to severe morbidity (e.g., amputation) and mortality [4,18]. In recent decades, electrotherapy was employed either with the use of contact electrodes or with the exposure to current flow, and has been applied for the ailment of chronic ulcers, as an alternative to biochemical compounds. ES is a safe, cost-effective and painless wound healing method that mimics the natural “current of injury” [42]. According to various studies, ES stimulates many cell types involved in wound or fracture healing, promoting their migration, proliferation and DNA synthesis, as well as the upregulation of TGF-β transcription, which mediates the synthesis of ECM proteins [17,43,44]. It has been shown that in response to exogenous EFs, keratinocytes, macrophages, epidermal and epithelial cells migrate to contribute to the wound closure [45,46,47]. Moreover, the ES of low amplitude induces angiogenesis both directly, through the activation of endothelial cells and indirectly, by stimulating the production of VEGF from various cell types [24,48]. The present study outlines the effects of microcurrents, which is another type of electrotherapy, in the cell types participating in the healing process.

Data from clinical cases suffering from chronic ulcers showed that treatment with microcurrents resulted in wound closure at 90–95% of the ulcer size. The treatment duration and the number of sessions required to achieve wound healing was found to be proportional to the severity of the injury [23,49]. Based on these data, we aimed to identify the molecular mechanisms underlying the microcurrent-stimulated healing process by using two different cell lines participating in wound and fracture healing: fibroblasts and osteoblast-like cells. The induction of cellular proliferation and migration via the activation of MAPKs ERK 1/2 and p38 is suggested as a possible mechanism by which microcurrents exert their action. These MAPKs are activated in response to a variety of extracellular stimuli, and regulate crucial cellular processes such as cell proliferation, differentiation and migration [50,51]. Moreover, it has been reported that the application of EFs triggered the phosphorylation of MAPKs ERK 1/2 and p38, in vitro [13]. In our study, in order to generate microcurrents, we used a commercially available WMCS device. NIH3T3 and MG-63 cell lines were stimulated with five different amounts of charges of −414, −916, −1672 and −3100 μC O_2_^−^, which did not induce cytotoxic effects. Our results verified that MAPKs ERK 1/2 and p38 are activated upon microcurrent stimulation, while maximal ERK 1/2 and p38 phosphorylation was observed when −916 μC O_2_^−^ and −414 μC O_2_^−^ were transferred to NIH3T3 and MG-63 cells, respectively. In accordance to previous studies, we proved that ERK 1/2 and p38 phosphorylation upon stimulation with microcurrents is specific, as indicated with the use of specific inhibitors for these kinases [50].

As aforementioned, it is well established that the activation of MAPKs ERK 1/2 and p38, through phosphorylation, induces cell migration and proliferation, whilst both of these cellular responses are involved in the wound healing process [51]. To address whether microcurrent stimulation enhances wound closure through cellular migration and/or proliferation, we applied a scratch-wound and cell proliferation assay on NIH3T3 and MG-63 cell lines. A highly significant increase in both the wound closure and proliferation rate was observed in the microcurrent-stimulated cells. Furthermore, these effects were dependent on the activation of MAPKs ERK1/2 or p38 in both cell lines, suggesting that microcurrent-induced cellular proliferation and migration is mediated by MAPKs signaling.

It is worth mentioning that during the exposure to microcurrents, the temperature of the culture medium was monitored and was found to be constantly stable. Thus, we excluded the possibility that the WMCS device generates heat, which could delay the wound healing process by reducing the re-epithelialized area [52]. As a result, the effect of microcurrents is not affected by the heat.

TGF-β plays a major role in the healing process by stimulating the proliferation of fibroblasts and induces the synthesis of proteins which constitute the ECM [19]. Our results indicate that treatment with microcurrents promotes the secretion of TGF-β1 in NIH3T3 and MG-63 cells, which is consistent with results from other studies where ES-induced TGF-β1 secretion in human fibroblasts [19]. Furthermore, according to our results, microcurrents induce TGF-β1 release in an ERK 1/2- and p38-dependent way. Except from affecting the activation of MAPKs signaling pathways, we examined whether treatment with microcurrents could, also, alter the transcriptome profile of osteoblasts. RNA seq revealed that from the 1139 genes analyzed; 121 were upregulated, 202 were downregulated and 816 were not differentially expressed in the MG-63 cells treated with −414 μC O_2_^−^ compared to the controls. Gene annotation ontology analysis uncovered that several upregulated genes participate in the TGF-β, MAPK or Hh signaling pathways in the MG-63 cells. On the other hand, downregulated genes participate mostly in metabolic pathways, such as the metabolism of purines and pyrimidines, which are involved in many biochemical processes, including DNA and RNA synthesis. Similarly, we detected the upregulation of genes which are involved in TGF-β and Hh signaling cascades in NIH3T3 cells. The Hh signaling pathway was identified as a novel target, which is activated by stimulation with microcurrents in both cell lines and thus, may enhance wound healing. Indeed, research data support that the Hh pathway is involved in the healing process by inducing cell proliferation, migration and angiogenesis [39,53].

To summarize, our data show that ES, in the form of microcurrents, promotes the activation of ERK 1/2 and p38 in the cell types implicated in wound or fracture healing, increases the expression levels of *Col1A1* and *Mmp19*, which are critical molecules for ulcers’ healing and upregulates TGF-β1, MAPKs and Hedgehog signaling pathways. Therefore, we confirmed that stimulation with microcurrents accelerates the healing process in vitro by triggering the phosphorylation of MAPKs ERK 1/2 and p38 and alters the transcriptome profile of the cells. Herein, we describe a simple experimental setup for the study of microcurrent stimulation effects in different cell lines and the identification of signaling pathways involved in the wound-healing process in vitro. Our study provides valuable insights in the mechanistic function of microcurrents. However, further investigation using different cell types, such as macrophages or keratinocytes, is needed. Furthermore, in vivo studies or experiments on human samples or organoids are required to verify the beneficial effects of microcurrent stimulation. Moreover, it is of high significance that future studies will eliminate the possibility of the persistent activation of MAPKs, Hh and TGF-β signaling pathways, or the downregulation of genes that are implicated in the metabolism during the treatment with microcurrents, as these pathways may be associated with tumorigenic phenotypes and severe adverse effects [54,55]. Indeed, the p38 signaling pathway has been shown to enhance prolonged inflammation in wounds [56].

## Supplementary Materials

The following are available online at https://www.mdpi.com/2073-4409/9/9/1924/s1, Figure S1: Experimental setup, temperature and pH values, MTT assay, Figure S2: Specificity of the U0126 and SB203580 inhibitors, Figure S3: Heatmap of the differentially expressed genes between the stimulated with microcurrents and the control cells, Figure S4: Gene ontology analysis of the downregulated genes, Figure S5: Transcriptional activation of the genes following the stimulation with microcurrents, Table S1: RNA seq results, Table S2: Gene annotation ontology analysis, Table S3: Upregulated and downregulated genes, Table S4: Table containing the sequences of primers used in this study.
