# Supplementary material for: Microcurrent Stimulation Triggers MAPK Signaling and TGF-β1 Release in Fibroblast and Osteoblast-Like Cell Lines
Source: Cells. 2020 Aug 19;9(9):1924. doi: 10.3390/cells9091924 (PMC7564311; doi:10.3390/cells9091924)
Supplement: Supplementary file 1 [file cells-09-01924-s001.zip › supplementary files/Supplementary Figures.pdf]

Supplementary Figures for article titled:

Microcurrent stimulation triggers MAPK signaling and TGF-β1 release in fibroblast and osteoblast-like cell lines

Evangelia Konstantinou, Zoi Zagoriti, Anastasia Pyriochou and Konstantinos Poulas*

Laboratory of Molecular Biology and Immunology, Department of Pharmacy, University of Patras, 26504 Rio, Greece

*Correspondence: kpoulas@upatras.gr

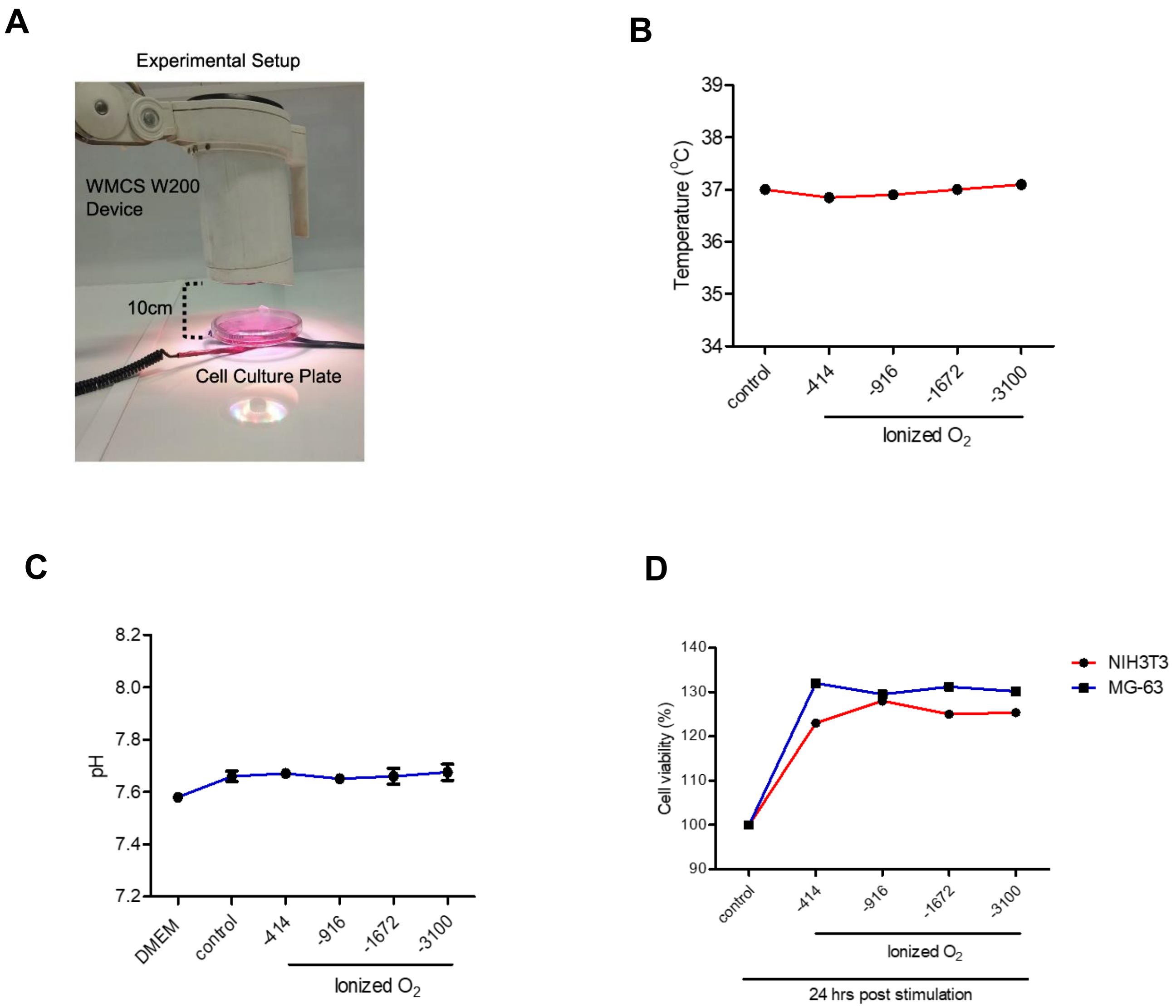

Figure S1. **A,** Experimental setup of cells stimulated with microcurrents using the WMCS W200 device. **B,** Temperature values in control and experimental cells following transfer of different charges. **C,** pH values of the medium, without any of cells, as well as from control and microcurrent stimulated cells. **D,** MTT assay, 24 hrs after the transfer of different charges in NIH3T3 and MG-63 cells as well as in control cells (N=3).

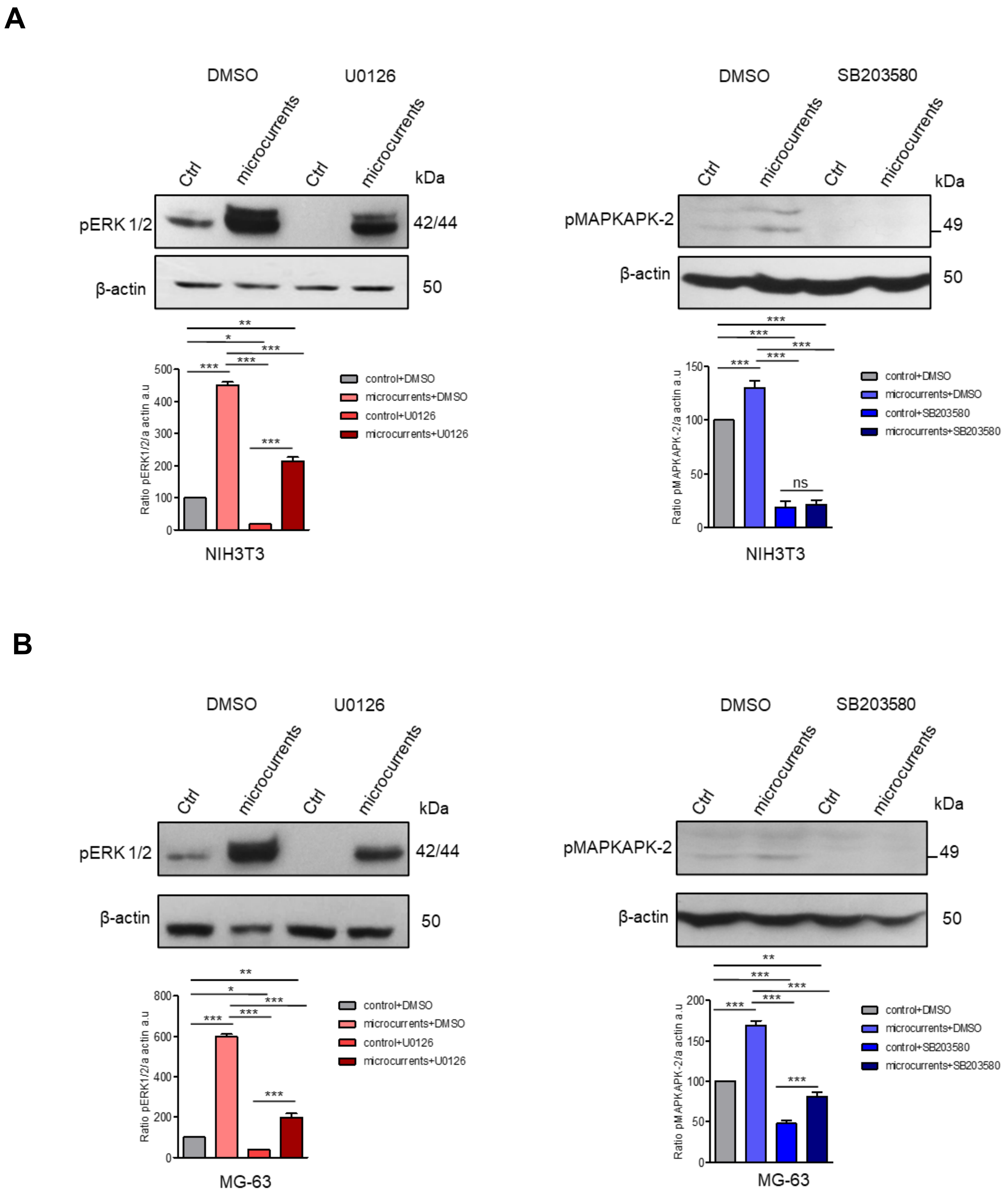

Figure S2. Specificity of U0126 and SB203580 inhibitors. Protein extracts from stimulated and control cells treated or not with inhibitors U0126 and SB203580 were analyzed with western blot. Western blot for pERK1/2 and pMAPKAPK-2 from protein extracts from stimulated **A,** NIH3T3 [Statistical analysis from all pairs of columns: control+DMSO vs microcurrents+DMSO (***), control+DMSO vs control+U0126 (*), control+DMSO vs microcurrents+U0126 (**), microcurrents+DMSO vs control+U0126 (***), microcurrents+DMSO vs microcurrents+U0126 (***), control+U0126 vs microcurrents+U0126 (***), control+DMSO vs control+SB203580 (***), control+DMSO vs microcurrents+SB203580 (***), microcurrents+DMSO vs control+SB203580 (***), microcurrents+DMSO vs microcurrents+SB203580 (***), control+SB203580 vs microcurrents+SB203580 (ns)] and **B,** MG-63 cells [Statistical analysis from all pairs of columns:

control+DMSO vs microcurrents+DMSO (***), control+DMSO vs control+U0126 (*), control+DMSO vs microcurrents+U0126 (**), microcurrents+DMSO vs control+U0126 (***), microcurrents+DMSO vs microcurrents+U0126 (***), control+U0126 vs microcurrents+U0126 (***), control+DMSO vs control+SB203580 (***), control+DMSO vs microcurrents+SB203580 (**), microcurrents+DMSO vs control+SB203580 (***), microcurrents+DMSO vs microcurrents+SB203580 (***), control+SB203580 vs microcurrents+SB203580 (***)]. Cells were incubated in the presence or absence of U0126 and SB203580 respectively. β-actin protein levels are shown as a loading control. Blots are representatives of 3 different experiments. (*p<0.05, **p<0.01, ***p<0.005, ns: no statistically significant, N=3, a.u: arbitrary units). Statistical analysis was performed with one-way ANOVA with Bonferroni's correction.

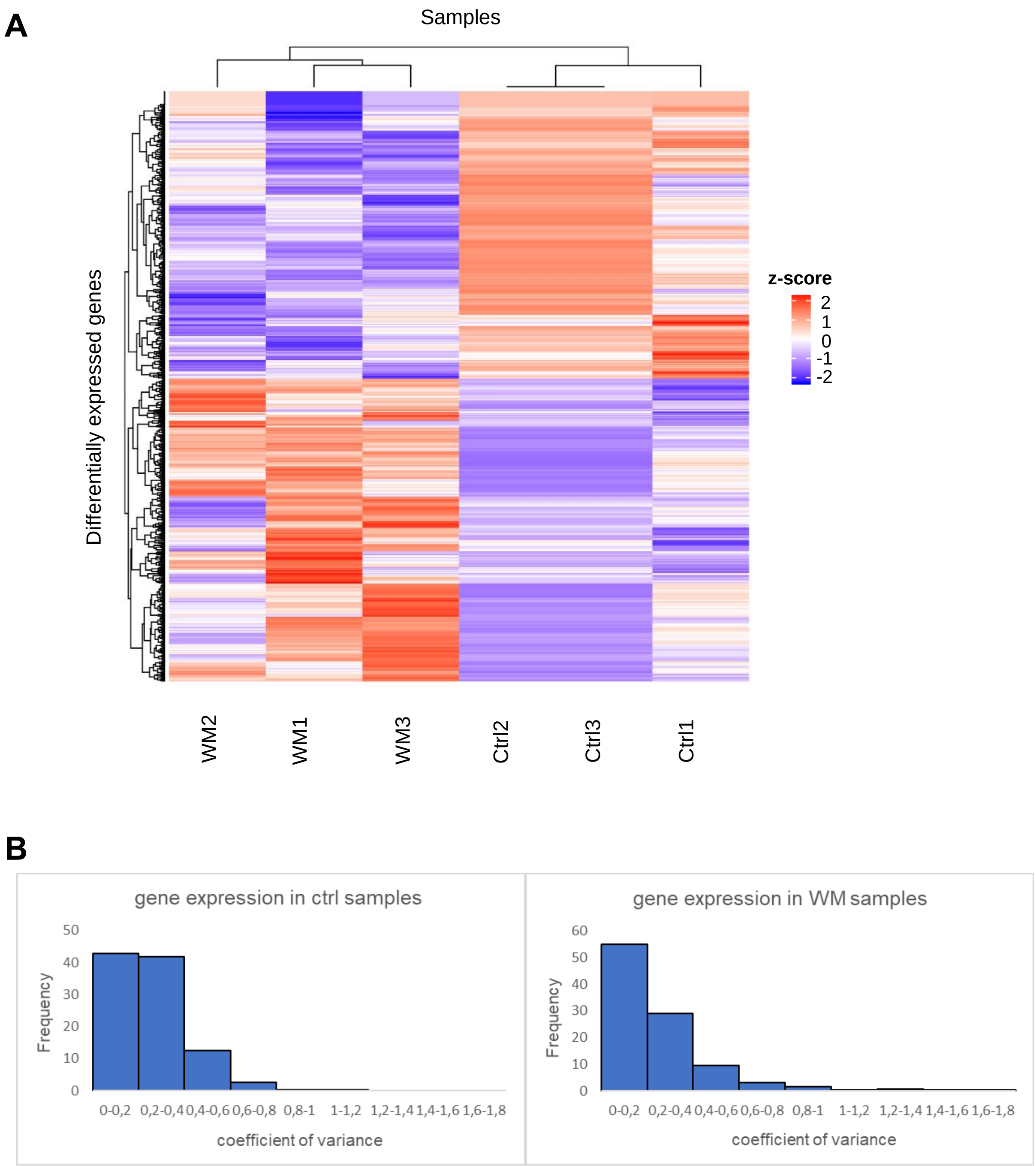

Figure S3. **A,** Heatmap of differentially expressed genes between stimulated with microcurrents and control MG-63 cells. Normalized read counts of genes are shown. Read counts are scaled per column. Red denotes high expression of genes, while blue denotes low expression. **B,** The genes from both control and stimulated cells, were grouped according to coefficient of variance. As shown in the graphs, the values for the majority of the genes tend to 0 (WM: cells stimulated with wireless microcurrents).

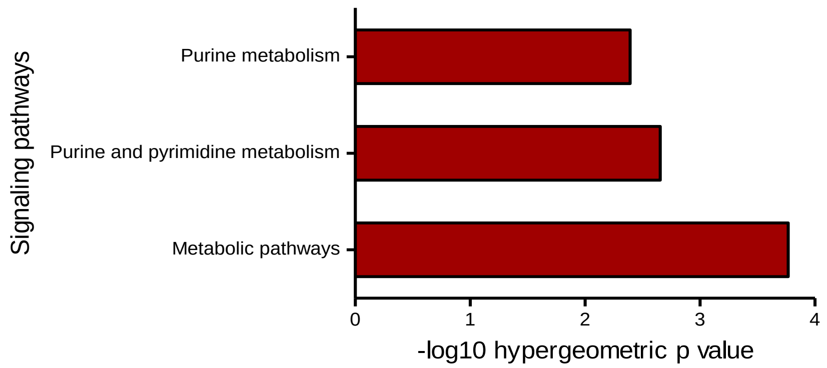

Figure S4. Gene ontology analysis of downregulated genes using GeneCodis.

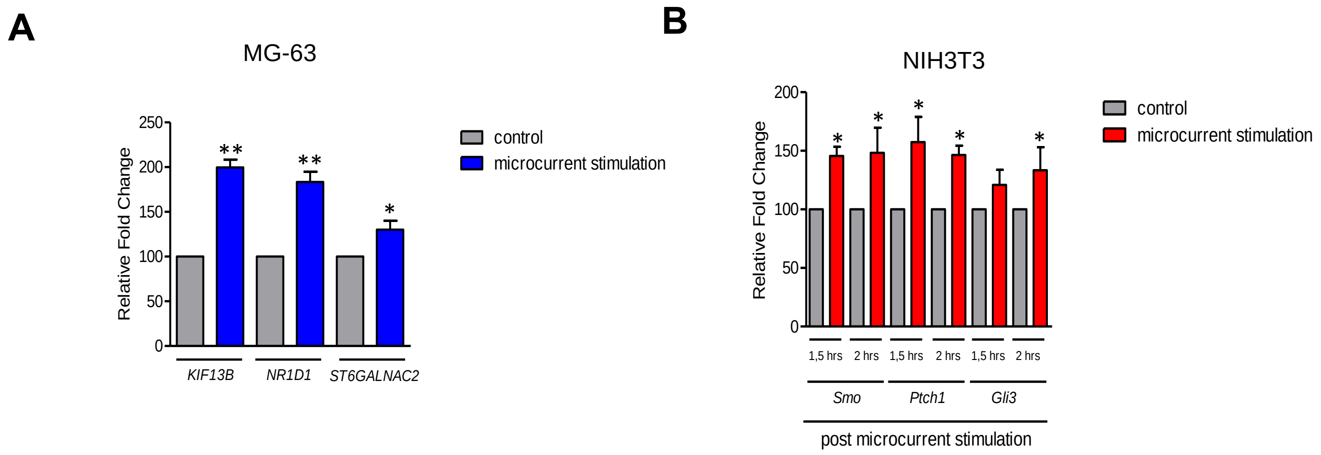

Figure S5. Transcriptional activation of genes following stimulation with microcurrents. **A,** Real Time PCR analysis for genes *NR1D1*, *KIF13B* and *ST6GALNAC2* in MG-63 cells, 8hr following stimulation with microcurrents. **B,** Real Time PCR analysis for *Smo*, *Ptch1* and *Gli3* genes in NIH3T3 cells, 1.5 and 2hr upon microcurrent stimulation. (*p<0.05, **p<0.01, treated vs control, n=3, N=3).
